# Case Report: Bronchoscopy-associated negative-pressure pulmonary edema in a child with severe *Mycoplasma pneumoniae* pneumonia

**DOI:** 10.3389/fped.2026.1946266

**Published:** 2026-09-09

**Authors:** Yang Wang, Yufan Zhang, Jun'e Li, Yuanyuan An, Le Wang, Yakun Wang

**Affiliations:** 1 Hebei Children's Hospital, Shijiazhuang, Hebei, China; 2 Hebei Clinical Research Center for Children's Health and Diseases, Shijiazhuang, Hebei, China; 3 Xinle Hospital, Xinle, Hebei, China

**Keywords:** airway hyperreactivity, bronchoscopy, negative pressure pulmonary edema (NPPE), pediatrics, severe *Mycoplasma pneumoniae* pneumonia

## Abstract

**Objective:**

To describe the clinical features, underlying mechanisms, and treatment approaches for negative pressure pulmonary edema (NPPE) that develops after flexible bronchoscopy (FB) in children with severe Mycoplasma pneumoniae pneumonia (SMPP).

**Methods:**

We retrospectively reviewed the case of a pediatric patient with SMPP who had NPPE during FB.

**Results:**

During bronchoscopy, the patient began coughing violently. Soon after, when the bronchoscope was in place, the patient showed signs of diffuse airway spasm. The Forceful inspiration against a functionally blocked airway led to a sharp drop in intrathoracic pressure. It triggered acute NPPE, severe hypoxemia, and acute respiratory failure. There were also biochemical evidence of myocardial injury and transient cardiac dysfunction. After emergency intubation with a 4.5 mm endotracheal tube and the start of mechanical ventilation, oxygenation improved quickly. The pulmonary edema largely resolved within 72 h. The patient was discharged on day 16.

**Conclusion:**

Children with SMPP tend to have marked airway hyperresponsiveness. This makes them more likely to have airway obstruction during bronchoscopy. When a child suddenly develops hypoxemia, significant dyspnea, and pink frothy sputum, NPPE should be strongly suspected. Careful selection of surgical timing and anesthesia strategy, continuous respiratory monitoring, timely relief of airway obstruction, and early positive pressure ventilation are crucial. It is also important to prepare for a difficult airway.

## 1 Background

Negative pressure pulmonary edema (NPPE) is an uncommon but potentially life-threatening form of non-cardiogenic pulmonary edema. It usually occurs in the setting of upper airway obstruction. What happens is that forceful inspiratory efforts against a blocked airway create markedly negative intrathoracic pressures, and this in turn drives fluid into the alveolar spaces (1). To date, no comprehensive statistical data have been published on the overall incidence of NPPE. A study of children with severe upper airway obstruction reported an NPPE incidence of 9.6% (2). Tami et al. observed an even higher rate of 12% in patients with acute upper airway obstruction (3). According to the Australian Incident Monitoring Study (AIMS), NPPE occurred in 3% of laryngospasm cases (4), whereas other literature reported a lower incidence of 0.1% in similar scenarios (5). No published literature has confirmed that missed diagnosis or inappropriate clinical interventions increase mortality risk, but these factors may elevate the incidence of shock, acute respiratory distress syndrome (ARDS), and acute kidney injury (AKI) (6).

Bronchoscopy-associated NPPE characteristically manifests as sudden respiratory distress, oxygen desaturation, and pink frothy sputum production during or immediately following acute airway obstruction. Chest imaging typically reveals newly emerged bilateral pulmonary opacities consistent with pulmonary edema (7, 8). When the airway obstruction is relieved promptly and positive-pressure ventilation is started, both respiratory symptoms and radiographic findings usually resolve within 24–48 h.

We report a pediatric case of SMPP who developed NPPE during FB. A laryngeal mask airway served as a rescue device to sustain oxygenation during interhospital transport, but definitive airway management was not achievable. This led to acute respiratory failure accompanied by biochemical markers of myocardial injury and transient cardiac strain. Documented cases of bronchoscopy-associated NPPE in pediatric patients with SMPP are very rare. In this case report, we highlight its clinical presentation, potential precipitating factors, and key principles of airway management.

## 2 Case presentation

### 2.1 Clinical course before transfer

**Figure 1:**
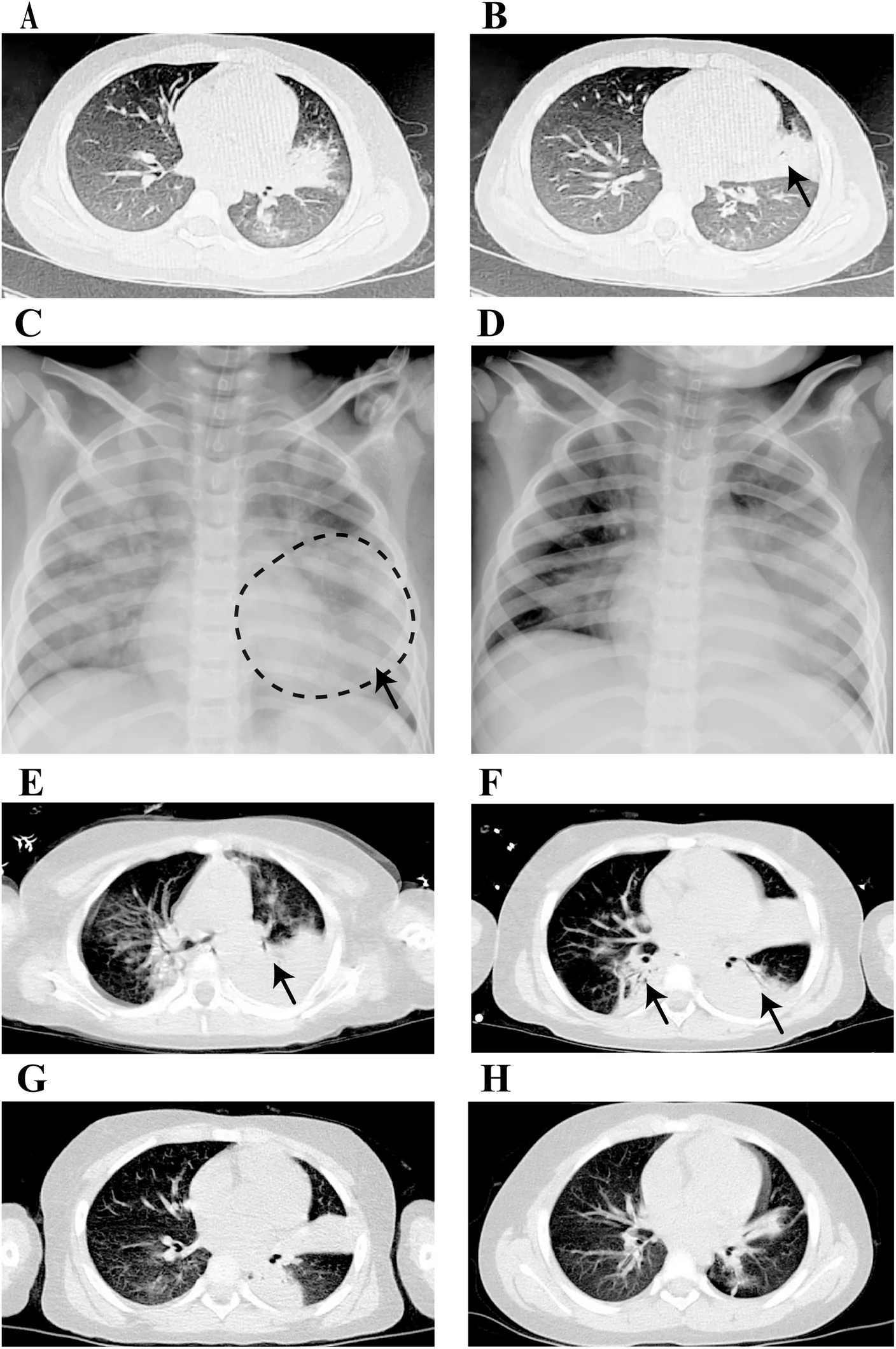
**(A,B)** chest CT before the first bronchoscopy showed inflammation and high-density opacities in the left lung, with a small amount of air bronchograms visible internally (arrows). **(C)** Immediate postoperative chest imaging revealed bilateral diffuse exudative opacities with left lung consolidation (arrows). **(D)** On postoperative day 3, the exudative opacities showed significant resolution. **(E,F)** Chest CT on day 6 after transfer demonstrated left upper lobe consolidation involving more than two-thirds of the lobe with distal bronchial obstruction, as well as partial consolidation in both lower lobes with air bronchograms (arrows). **(G)** Pre-discharge imaging revealed only focal consolidation in the left lung. **(H)** Follow-up CT showed near-complete resolution of the consolidative opacities.

A 5-year-old boy was admitted to Xinle Municipal Hospital. He had a two-day of fever and cough. His highest temperature was 39.5 °C., and he weighed 26 kg. There was no prior history of wheezing, allergic conditions, congenital airway anomalies, or any previous exposure to bronchoscopy or anesthesia. Initial blood work showed an elevated white blood cell count (15.44 × 10⁹/L) with neutrophil predominance (82.8%), and a marked rise in C-reactive protein (61.31 mg/L). This pointed to a strong inflammatory response. Lymphocytes were on the lower side (10.2%). Coagulation studies were essentially normal except for a mildly elevated D-dimer (0.52 mg/L). Other lab tests showed increased lactate dehydrogenase (359 U/L) and *α*-hydroxybutyrate dehydrogenase (296 U/L), and an accelerated erythrocyte sedimentation rate (40 mm/h). Mycoplasma pneumoniae DNA testing came back positive. A chest CT scan confirmed left lower lobe consolidation with suspected mucus plug-induced atelectasis (Figure 1). Based on these findings and the Chinese Guidelines for the Diagnosis and Treatment of Mycoplasma pneumoniae Pneumonia in Children (2025 Edition), he was diagnosed with SMPP. Treatment was started right away with azithromycin combined with intravenous methylprednisolone.

Because of the clinical suspicion of left lower lobe atelectasis from mucus plug obstruction, bronchoalveolar lavage via flexible bronchoscopy was planned for the fifth day of illness. The patient was kept NPO for 8 h before the procedure. About 15 min before the procedure, he received nebulized 2% lidocaine. Pre-procedure vitals were stable. Breath sounds over the left lower lung field were diminished on auscultation. He was on nasal cannula oxygen with a recorded temperature of 36.8 °C.

For conscious sedation, we used IV midazolam (7.5 mg bolus). A 3.7 mm flexible scope was passed transnasally. Then we used titrated topical anesthesia with 2% lidocaine (total 8.5 mL, about 6.5 mg/kg),which delivered through the bronchoscope. We started with the upper airways, and moved down the tracheobronchial tree to the segmental level. Our team have one operator and two nurses, keeping to a strict protocol. One person advanced the scope while the others tracked vitals and medication effects. Oxygen was administered via nasal cannula throughout the bronchoscopy procedure.

Initial inspection showed right bronchial mucosal erythema and minimal secretions. When the bronchoscope reached the bifurcation of the left main bronchus, the patient suddenly developed severe coughing. Within about five seconds, perioral cyanosis appeared, accompanied by significant tachycardia. Peripheral oxygen saturation decreased rapidly. The scope was withdrawn immediately. For suspected tracheal spasm, nebulized inhalation of bronchodilator, salbutamol 2.5 mg, combined with ipratropium bromide 0.25 mg, was given. Intravenous dexamethasone 3 mg was also administered to reduce airway inflammatory edema. However, the patient's oxygen saturation remained at around 70% for about 40 s.

First attempt to intubate with a 5.5 mm uncuffed tracheal tube failed. This attempt failed. Laryngoscopy showed a narrowed glottic aperture. Then a size 2.5 laryngeal mask airway was placed immediately as a rescue airway to secure ventilation. Soon after positive pressure ventilation was started, the patient began producing pink frothy sputum. This was a clear sign of acute pulmonary edema. Emergency IV medications were given. 15 mg of methylprednisolone sodium succinate was administered to suppress alveolar exudation and bronchospasm, 15 mg of furosemide was given to reduce pulmonary circulatory overload. And 45 mg of mannitol was used to help alleviate interstitial pulmonary edema.

Within two minutes of laryngeal mask ventilation combined with these medications, the patient’s peripheral oxygen saturation improved to roughly 94%, with a recorded body temperature of 37 °C.

Emergency arterial blood gas analysis revealed: pH 7.21, PaO₂ 94 mmHg,SpO₂ 97.1%, PaO₂/FiO₂ ratio 448 mmHg, PaCO₂ 61 mmHg, base excess −4.5 mmol/L, and bicarbonate 24.1 mmol/L.At the same time, a complete blood count showed marked leukocytosis (20.14 × 10⁹/L) with neutrophilia (83.8%, absolute count 25.26 × 10⁹/L), lymphopenia (12.7%, absolute count 3.83 × 10⁹/L), and an elevated C-reactive protein (47.55 mg/L). Once vital signs were stabilized, the patient was urgently transferred to Hebei Children's Hospital and admitted to the Pediatric Intensive Care Unit within 30 min. (Table 1).

**Table 1:** Timeline of acute airway emergency complicated with negative-pressure pulmonary edema during flexible bronchoscopy.

Time point (from 0 s)	Core interventions	Clinical signs
0 s	Bronchoscope advanced to the left main bronchus bifurcation	Sudden severe cough, rapid onset of perioral cyanosis, significant tachycardia, and sharp decline in SpO₂
5 s	1. Immediate full withdrawal of the bronchoscope 2. Nebulized bronchodilators (salbutamol 2.5 mg + ipratropium bromide 0.25 mg) 3. Intravenous dexamethasone 3 mg	SpO₂ persisted at ∼70% for 40 consecutive seconds with no improvement
5∼45 s	1. Failed endotracheal intubation 2. Emergency placement a LMA 3. Intravenous medications: methylprednisolone sodium succinate 15 mg, furosemide 15 mg, mannitol 45 mg	Pink frothy sputum
Within 2 min	Continuous LMA-assisted ventilation and vital sign monitoring	SpO₂ improved to 94%; recorded body temperature 37 °C
After vital sign stabilization	1. Emergency ABG and CBC sampling 2. Initiation of urgent inter-hospital transfer	ABG: pH 7.21, PaO₂ 94 mmHg, PaO₂/FiO₂ 448 mmHg, PaCO₂ 61 mmHg, BE −4.5 mmol/L CBC: WBC 20.14 × 10⁹/L, NE% 83.8%, CRP 47.55 mg/L
30 min	Completion of inter-hospital transfer	Admitted to ICU

### 2.2 Management after transfer

On arrival in the PICU, the patient was still being supported with a laryngeal mask airway for oxygenation, and his non-invasive blood pressure was 98/60 mmHg. On exam, he had perioral cyanosis along with tachypnea and nasal flaring, plus obvious suprasternal, intercostal, and subcostal retractions. Cardiovascular exam showed tachycardia with muffled heart sounds. On auscultation of the lungs, there were bilateral rales and wheezing. Physical examination revealed nail bed cyanosis. The extremities were warm, and the capillary refill time was approximately three seconds. An emergency arterial blood gas analysis revealed pH 7.385, PaCO₂ 42.5 mmHg, PaO₂ 57.2 mmHg, HCO₃⁻ 25.7 mmol/L, base excess −1.1 mmol/L, SpO₂ 88.3%, and a PaO₂/FiO₂ ratio of 273.9 mmHg, with hemoglobin at 12.8 g/dL. The aforementioned examination results and clinical manifestations confirmed the presence of acute respiratory failure in the patient. Seek immediate consultation from the anesthesiology department. A 4.5 mm non-cuffed endotracheal tube was used to establish an artificial airway. Pressure-controlled ventilation was initiated. The settings were FiO₂ 80%, PEEP 8 cmH₂O, respiratory rate 28 breaths/min, and driving pressure 16 cmH₂O above PEEP. Administer midazolam for sedation.

Blood tests showed a lactate of 1.2 mmol/L, serum myoglobin 113.99 ng/mL, creatine kinase-MB 5 ng/mL, and cardiac troponin I 0.309 ng/mL. NT-proBNP was notably elevated at 557.3 pg/mL. The electrocardiogram indicated sinus tachycardia. The echocardiogram showed preserved cardiac function. The left ventricular ejection fraction was 65%, with no structural abnormalities observed. The patient had no previous history of heart disease and no signs of heart failure. Therefore, the elevated myocardial markers were caused by stress-induced myocardial injury due to pulmonary lesions. The bedside chest x-ray revealed new bilateral diffuse pulmonary infiltrates. This was consistent with the presentation of pulmonary edema on the basis of pre-existing pneumonia, without cardiac enlargement (Figure 1).

During bronchoscopy, the patient suddenly developed intense paroxysmal coughing, severe hypoxemia, and pink frothy sputum. Direct laryngoscopy showed glottic stenosis. Considering the above manifestations and previous diagnostic results, it is consistent with negative pressure pulmonary edema accompanied by acute respiratory failure. It is also consistent with transient myocardial injury and increased cardiac load.

To reduce myocardial load, the child was administered milrinone and digoxin. Milrinone could rapidly provide positive inotropic support and reduce afterload by dilating blood vessels. Digoxin was used to continuously enhance myocardial contractility. After the aforementioned treatment, the perioral and nail bed cyanosis subsided. A repeat arterial blood gas showed clear improvement: pH 7.43, PaCO₂ 33.2 mmHg, PaO₂ 248.9 mmHg, HCO₃⁻ 22.3 mmol/L, base excess −0.8 mmol/L, SpO₂ 99.9%, and a markedly elevated PaO₂/FiO₂ ratio of 553.1 mmHg. Hemoglobin was 11.9 g/dL. Additionally, azithromycin was administered for anti-infective treatment.

On the third day after transfer, the pulmonary edema had clearly improved both clinically and radiographically, but extensive parenchymal consolidation was still present (Figure 1). On day 6 after transfer, a follow-up chest CT showed progressive consolidation in the left lower lobe, new consolidative lesions in the left upper lobe, and air bronchograms in both lower lobes (Figure 1). Because of these radiographic findings and their possible link to airway obstruction, repeat flexible bronchoscopy with bronchoalveolar lavage was recommended.

On day 8 after transfer, the patient underwent flexible bronchoscopy with bronchoalveolar lavage through the endotracheal tube under standard sterile conditions. Before the procedure, the child was sedated with midazolam. During this time, the existing endotracheal tube was exchanged for a 5.0-mm uncuffed one. Thirty minutes before the bronchoscope was inserted, 0.26 mg atropine was given intramuscularly to reduce secretions and blunt vagal responses. Pre-oxygenation was given just before the bronchoscope was inserted. A 2.8-mm flexible bronchoscope was then introduced through the indwelling endotracheal tube. Vital signs were monitored continuously throughout.

**Figure 2:**
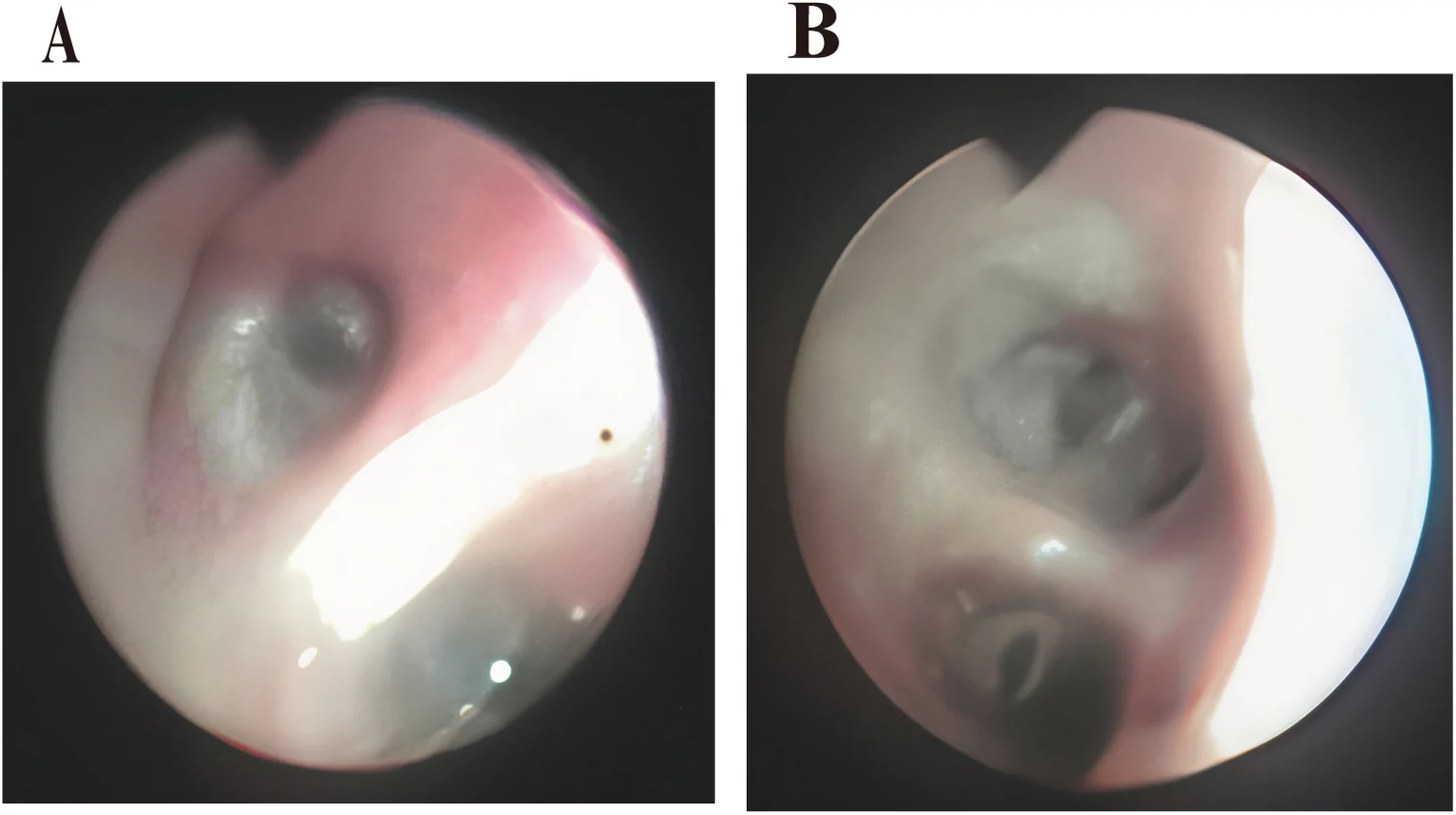
Follow-up bronchoscopy on day 8 post-transfer revealed pale, wrinkled mucosa. **(A)** Abundant flocculent secretions were noted at the segmental orifices of the left upper lobe. **(B)** Flocculent and cord-like secretions were observed in the basal segments of the right lower lobe.

The bronchoscopy showed rough, pale, and wrinkled bronchial mucosa. White flocculent secretions were seen in moderate amounts mainly in the lingular segments of the left upper lobe and the anterior basal segment of the right lower lobe. No mucus plugs were found at any point during the procedure (Figure 2).

Metagenomic next-generation sequencing (mNGS) of the BAL fluid showed dominant sequence reads for Mycoplasma pneumoniae (47,016 reads, relative abundance 34.5%). This was followed by Fusobacterium nucleatum (19,967 reads, 14.7%) and Prevotella oulorum (13,049 reads, 9.6%). Low-abundance reads for Pseudomonas aeruginosa were also found (74 reads, relative abundance 0.054%).

The mNGS results showed Mycoplasma pneumoniae was the dominant organism. It had the highest read count and relative abundance. The patient's clinical presentation, the consolidative opacities on chest CT, and the earlier positive peripheral blood nucleic acid test for M. pneumoniae all pointed to this pathogen as the primary cause of the pulmonary infection. Fusobacterium nucleatum and Prevotella oulorum are typical upper oral commensals. They were likely detected because of oropharyngeal colonization that contaminated the bronchoscopic specimen. For Pseudomonas aeruginosa, the read numbers were minimal and the relative abundance was low. The sequencing profile suggested it had moderate pathogenic potential. The patient had repeated endotracheal intubation, so the clinical team stayed alert for possible secondary pulmonary infection and clinical deterioration. They started additional antimicrobial coverage with meropenem.

### 2.3 Clinical outcome

After these treatments, the patient improved markedly, and the pulmonary findings on serial imaging also regressed. Follow-up chest CT scans showed significant resolution of the previously noted consolidations, and the air bronchograms had disappeared (Figure 1). The patient met discharge criteria and was released on hospital day 16 post-transfer.

### 2.4 Follow-up

At the 69-day post-discharge follow-up, the patient remained asymptomatic with clear bilateral lung fields on auscultation. Follow-up chest CT imaging demonstrated nearcomplete resolution of the previously noted pulmonary consolidation lesions (Figure 1).

## 3 Discussion

Negative pressure pulmonary edema is a rare type of non-cardiogenic pulmonary edema that can lead to human death. Its core pathogenesis is the generation of extremely high intrathoracic negative pressure during forced inhalation when the airway is obstructed. The markedly elevated negative intrathoracic pressure enhances systemic venous return while simultaneously decreasing perivascular interstitial hydrostatic pressure and abruptly increasing left ventricular afterload. These hemodynamic changes cause fluid to move across pulmonary capillary membranes into the interstitial spaces and alveoli, leading to pulmonary edema (9). Earlier reports show that NPPE is usually linked to anatomical airway obstruction, such as laryngospasm, foreign body aspiration, or post-anesthetic extubation (7, 8, 10–12). But in this case, a child with SMPP developed sudden NPPE during bronchoscopy. Airway hyperresponsiveness and generalized airway spasm caused by lower respiratory tract infection are unique risk factors for NPPE. Two key airway-management problems are also illustrated by this case. Emergency airway protocols were not enough. Wrong endotracheal tube sizes were used. These factors show the importance of strict airway safety management during bronchoscopy in high-risk children and provide a useful clinical reference for diagnosing and managing similar cases.

The patient had circumoral cyanosis, oxygen desaturation to 85%, and frothy pink-tinged sputum after acute respiratory distress. A bedside chest x-ray taken immediately showed bilateral diffuse alveolar infiltrates. These matched classical NPPE (6). The patient had followed the right fasting rules before the procedure for both solids and liquids. Clinicians carefully controlled IV fluids during the procedure. There were no clinical signs of gastric regurgitation during the procedure. Bronchoscopy showed no gastric contents in the airways. This ruled out aspiration or fluid overload as causes of the cough and pulmonary edema. Before the bronchoscopy, vital signs were stable throughout. There were no signs of worsening infection or high inflammatory markers. The bilateral pulmonary infiltrates cleared quickly within 72 h after starting positive pressure ventilation. This does't fit with infectious pulmonary injury or infection-associated ARDS. The patient had pink frothy sputum but no ongoing hemoptysis. Hemoglobin levels stayed normal. The patient improved without high-dose steroid pulse therapy or hemostatic agents. This ruled out diffuse alveolar hemorrhage. The patient had no heart disease before this. Preoperative cardiac auscultation was normal. There were no symptoms of heart failure. Normal cardiac structure was demonstrated by transthoracic echocardiography, making cardiogenic pulmonary edema unlikely. The elevations in NT-proBNP, MYO, CK-MB, and cTnI were due to secondary changes from the very high intrathoracic negative pressure. Additionally, NPPE usually appears acutely after upper airway obstruction. And, it usually improves a lot within 24–48 h after starting positive-pressure ventilation (9). In this case, the radiographic pulmonary edema had mostly cleared by the third day of mechanical ventilation. This strongly supports the NPPE diagnosis.

Airway hyperresponsiveness caused by infection probably makes these children more likely to develop procedure-induced airway spasm. Mycoplasma infection directly injures the respiratory epithelium. This starts a dysregulated immune response—bronchial wall edema, heightened smooth muscle hyperreactivity—and leads to marked airway hyperresponsiveness (13). One animal study found that both inflammatory responses and airway hyperreactivity peak on day 3 after infection, much higher than levels on day 7 (14). It is not known if the same timing happens in children with M. pneumoniae infection. More studies are needed, but it seems possible.

Repeat bronchoscopy showed no bronchial casts or mucus plugs. The first chest CT suggested possible atelectasis from mucus plugs, but the first bronchoscopy had to be stopped suddenly when pulmonary edema developed. So it is still not clear if mucus plugs were present during that first procedure.

Poor anesthesia management may also increase the risk of airway spasm (15). In children with SMPP, conscious sedation alone, without antispasmodic drugs, may not suppress airway reflexes enough.

Airway support for this patient failed on the first round of endotracheal intubation attempts, and only after the second attempt was a stable airway obtained. As this case, severe diffuse airway spasm can make it difficult to secure a definitive airway. After the development of generalized airway spasm, timely opening of the airway and application of effective positive-pressure ventilation are life-saving measures (9, 16). If standard endotracheal intubation is not possible, a laryngeal mask airway (LMA) can be used as a temporary rescue method for ventilation. However, it can't offer definitive tracheal airway control. In this case, the patient's percutaneous oxygen saturation dropped to 88.3% during interhospital transfer while on LMA ventilation. Therefore, clinicians should quickly replace the LMA with an appropriately sized endotracheal tube and consider bringing in an anesthesiologist if needed. Once positive-pressure ventilation is started through the endotracheal tube, it helps relieve spasm and stops the pulmonary edema from getting worse. See Supplementary Discussion for further detailed discussion of treatment.

## 4 Conclusions

In short, the management of this case provides important clinical information for perioperative care during bronchoscopy in children with SMPP. Such patients have airway hyperresponsiveness and mucosal oedema, and are thus at high risk of NPPE. It's worth incorporating individualized procedural timing, pre-procedure airway-risk assessment, appropriate anesthesia or sedation planning, continuous monitoring, and a predefined rescue-airway strategy. NPPE can cause sudden cyanosis and oxygen deprivation, and the expectoration of pink frothy sputum. The bronchoscope needs to be removed immediately to relieve the airway obstruction and positive-pressure ventilation should be started promptly to correct hypoxemia. Clinicians should rapidly adjust the airway strategy when endotracheal intubation fails. Ventilation can be carried out with LMAs, or a more appropriate endotracheal tube can restore oxygenation fast and Reduce the incidence of complications.

## Data Availability

The original contributions presented in the study are included in the article/Supplementary Material, further inquiries can be directed to the corresponding authors.
