# Supplementary material for: Case Report: Bronchoscopy-associated negative-pressure pulmonary edema in a child with severe Mycoplasma pneumoniae pneumonia
Source: Front Pediatr. 2026 Sep 9;14:1946266. doi: 10.3389/fped.2026.1946266 (PMC13597900; doi:10.3389/fped.2026.1946266)
Supplement: Supplementary file 1 [file Supplementaryfile1.pdf]

Supplementary Discussion

The use of diuretics is the subject of controversy. Traditionally, for patients diagnosed with NPPE, diuretics are recommended as an adjunct therapy [1,2]. However, some scholars argue that NPPE is a result of systemic fluid redistribution, rather than fluid overload. Indiscriminate use of diuretics may exacerbate the patient's condition, leading to hypovolemic shock [3,4]. A small number of patients may develop acute respiratory distress syndrome and acute kidney injury due to overuse or misuse of diuretics [5]. In this case, the child was fasting before surgery, and the use of diuretics carried risks. Although no severe complications occurred in this case, it still serves as a reminder that clinicians should carefully consider the use of diuretics.

Corticosteroids mainly have anti-inflammatory effects and cannot address the pathophysiology of NPPE [5]. However, since the child in this case had SMPP, the use of dexamethasone and methylprednisolone was more aimed at alleviating inflammation caused by infection, thereby reducing airway spasm and edema. The use of mannitol and inotropic drugs has not found research or guideline basis. Clinicians used mannitol early in this case because the child's condition was severe initially, and there was concern about the child developing cerebral edema. The possibility of cardiogenic pulmonary edema can be ruled out in this case. Based on the pathogenesis of pulmonary edema, the increase in NT-proBNP, MYO, CK-MB, and cTnI is a stress response caused by NPPE. The use of milrinone and dobutamine carries the risk of overtreatment.

Supplementary References

[1] Bhattacharya, M., Kallet, RH., Ware, LB., Matthay, MA, (2016). Negative-Pressure Pulmonary Edema. CHEST, 150 (4), 927-933. https://doi.org/10.1016/j.chest.2016.03.043

[2] Guru, PK., Agarwal, A., Pimentel, M., McLaughlin, DC., Bansal, V, (2018). Postoperative Pulmonary Edema Conundrum: A Case of Negative Pressure Pulmonary Edema. Case Rep Crit Care, 2018 1584134. https://doi.org/10.1155/2018/1584134

[3] Maxwell, BG., Mihm, FG, (2011). Questioning diuretic use in acute negative-pressure pulmonary edema. ANESTHESIOLOGY, 114 (2), 461. https://doi.org/10.1097/ALN.0b013e31820708c4

[4] Raj, D., Priti, K., Quari, H., Jha, R, (2016). An anesthetic management of negative pressure pulmonary edema CHRISMED J Health Res, 3 (3), 223. https://doi.org/10.4103/2348-3334.183750

[5] Shaikh N, Alali B, Amara UE, Nashrah UE, Ummunnisa F. Negative-Pressure pulmonary edema: a perioperative emergency. Cureus. (2025) 17(9):e91868. doi: 10. 7759/cureus.91868
